# Editorial Expression of Concern: birinapant sensitizes platinum-resistant carcinomas with high levels of cIAP to carboplatin therapy

**DOI:** 10.1038/s41698-018-0062-1

**Published:** 2018-08-01

**Authors:** V. La, R. Fujikawa, D. M. Janzen, M. Nunez, L. Bainvoll, L. Hwang, K. Faull, G. Lawson, S. Memarzadeh

**Affiliations:** 1 Department of Obstetrics and Gynecology, David Geffen School of Medicine, University of California Los Angeles, Los Angeles, CA 90095 USA; 2 Department of Psychiatry and Biobehavioral Sciences, David Geffen School of Medicine, University of California Los Angeles, Los Angeles, CA 90095 USA; 3 Pasarow Mass Spectrometry Laboratory, University of California Los Angeles, Los Angeles, CA 90095 USA; 4 Semel Institute for Neuroscience and Human Behavior, University of California Los Angeles, Los Angeles, CA 90095 USA; 5 Office of Laboratory Animal Care, University of California Berkeley, Berkeley, CA 94720 USA; 6 UCLA Eli and Edythe Broad Center of Regenerative Medicine and Stem Cell Research, University of California Los Angeles, Los Angeles, CA 90095 USA; 7 Molecular Biology Institute, University of California Los Angeles, Los Angeles, CA 90095 USA; 8 Department of Biological Chemistry, David Geffen School of Medicine, University of California Los Angeles, Los Angeles, CA 90095 USA; 9 The VA Greater Los Angeles Healthcare System, Los Angeles, CA 90073 USA

**Keywords:** Cancer therapeutic resistance, Predictive markers

**Addendum to:** *npj Precision Oncology*; 10.1038/s41698-017-0008-z; Published online 03 April 2017.

*npj Precision Oncology* is publishing an Editorial Expression of Concern on the manuscript ‘Birinapant sensitizes platinum-resistant carcinomas with high levels of cIAP to carboplatin therapy’ from La et al., because of a letter requesting retraction communicated to our journal by S. Memarzadeh, a corresponding author of this manuscript. Concerns have been raised regarding the identity of the cell lines used for the experimental work in Fig. 1d, and the reporting of the survival of cells presented in Fig. 2a. We understand that this is subject to an ongoing review, and we will continue to monitor the situation and update this statement accordingly. The authors have been informed of this statement. V. La, R. Fujikawa, M. Nunez, L. Bainvoll, L. Hwang, K. Faull and S. Memarzadeh agree with this editorial expression of concern. We were unable to obtain a response from D. M. Janzen and G. Lawson.

